# Case Report: Late choroidal metastasis from hormone receptor-positive, HER2-negative breast cancer responsive to first-line endocrine therapy

**DOI:** 10.3389/fonc.2026.1719671

**Published:** 2026-03-25

**Authors:** Hisham Alsharif, Robert Wesolowski, Colleen M. Cebulla, Ashley P. Davenport, Margaret E. Gatti-Mays, Kai C. C. Johnson, Nerea Lopetegui-Lia, Dionisia Quiroga, Arya Mariam Roy, Mohammad Shujaat, Daniel G. Stover, Gilbert Bader

**Affiliations:** 1 Division of Medical Oncology, Department of Internal Medicine, The Ohio State University Comprehensive Cancer Center, Columbus, OH, United States; 2 Department of Ophthalmology and Visual Sciences, The Ohio State University Wexner Medical Center, Columbus, OH, United States; 3 Department of Radiology, The Ohio State University Wexner Medical Center, Columbus, OH, United States

**Keywords:** breast cancer, case report, CDK4/6, choroidal metastasis, endocrine therapy

## Abstract

Distant metastatic breast cancer can occur years after initial diagnosis, with choroidal metastasis being a rare but significant manifestation. This case report presents a patient with a history of estrogen receptor-positive (ER+), human epidermal growth factor receptor 2-negative (HER2-) invasive ductal carcinoma (IDC) of the left breast, who developed late choroidal metastasis. The patient underwent systemic therapy with an aromatase inhibitor and cyclin-dependent kinase 4 and 6 (CDK4/6) inhibitor leading to regression of choroidal metastases. We also conducted a literature review of similar cases. External beam radiotherapy is the gold standard for management of choroidal metastases. However, it appears that first-line treatment with endocrine therapy and CDK4/6 inhibitors in patients with ER+ and HER2- breast cancer is likely effective for the treatment of choroidal metastases secondary to ER+/HER2- breast cancer and may allow a delay in the use of local invasive interventions.

## 1 Introduction

Metastatic lesions are the most common ocular malignant tumors. The prevalence of ocular metastases is estimated to be around 5-10% (1). Since the choroid is the only intraocular tissue that is located outside the blood-aqueous and blood-retinal barriers, choroidal metastases (CM) represent 90% of ocular metastases. Breast cancer is the primary source in 53% of cases (2). The median interval between breast cancer diagnosis and CM is 42.4 months, but cases occurring up to 34 years later have been reported (3, 4). CM are likely underdiagnosed since ocular examinations are not routinely performed in patients with metastatic cancer, even in those with mild ocular symptoms. Identification and treatment of CM is crucial since progression can lead to visual loss (5). We report a case of a 57-year-old female with metastatic estrogen receptor-positive (ER+), human epidermal growth factor receptor 2-negative (HER2-) breast cancer with choroidal, pulmonary, and osseous metastases who experienced an excellent response to first-line endocrine therapy in combination with a cyclin-dependent kinase 4 and 6 (CDK4/6) inhibitor. We also conducted a literature review of similar cases. Written informed consent was obtained from the patient for publication of this case report and any accompanying images.

## 2 Case description

A 57-year-old female with a remote history of ER+, progesterone receptor-positive (PR+), HER2- invasive ductal carcinoma (IDC) of the left breast, originally diagnosed in April 1994, presented in 2023 with sudden central vision loss in her left eye. At the time of her initial diagnosis, she underwent a left mastectomy and axillary lymph node dissection (ALND), with pathology showing pT2 pN0 disease. She was treated with adjuvant chemotherapy consisting of doxorubicin and cyclophosphamide but did not receive endocrine therapy for unknown reasons. An ophthalmologic examination in 2023 revealed an amelanotic choroidal lesion associated with macular edema. Subsequent computed tomography (CT) of the chest, abdomen, and pelvis demonstrated multiple pulmonary nodules, subpleural nodularity, mediastinal lymphadenopathy, and a small subcapsular hepatic lesion too small to characterize — findings concerning for metastatic recurrence of breast cancer.

**Figure 1:**
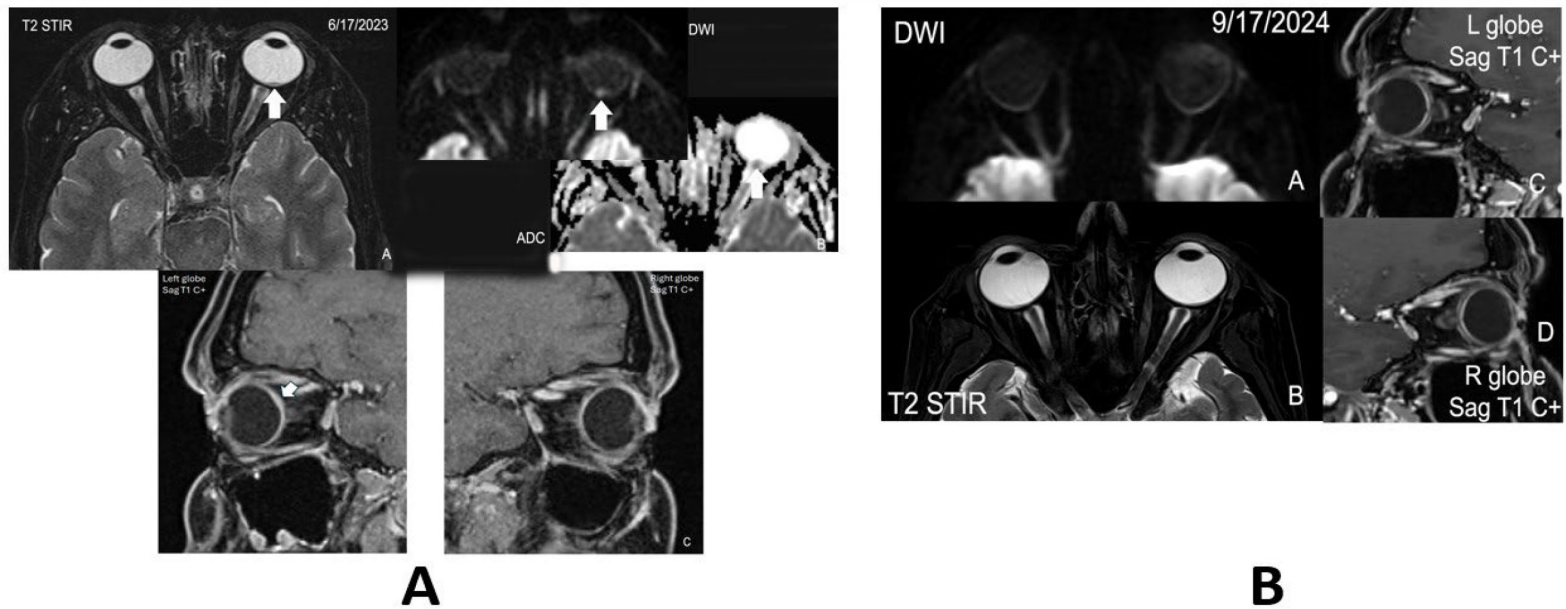
**(A)** (A-C). Orbit MRI at diagnosis. (A) Orbit MRI axial STIR/T2 fat sat images show a region of focal left choroidal thickening (white arrow). (B) Orbit MRI axial DWI and ADC images demonstrate the lesion near the optic disc with true restricted diffusion indicating cellularity. (C) Sag oblique T1 post-contrast 3D/volumetric VIBE sequences show a very subtle choroidal thickening not seen on any other sequences, more conspicuous compared to the normal right globe/choroid. **(B)** (A-D). Post-treatment orbit MRI images. Post-treatment orbit MRI with axial DWI (A), T2 STIR images (B) demonstrating resolution of the choroidal lesion with more symmetric uveoscleral layers after resolution of the focal left choroidal thickening seen previously on post-contrast images [white arrow, (C)] compared to the normal right globe (D).

Magnetic resonance imaging (MRI) of the orbits demonstrated subtle uveal thickening with possible retinal detachment/effusion in the left eye (**Figure 1**). MRI of the brain was unremarkable for intracranial metastasis. Given the visual symptoms and the known history of breast cancer, CM was suspected.

**Figure 2:**
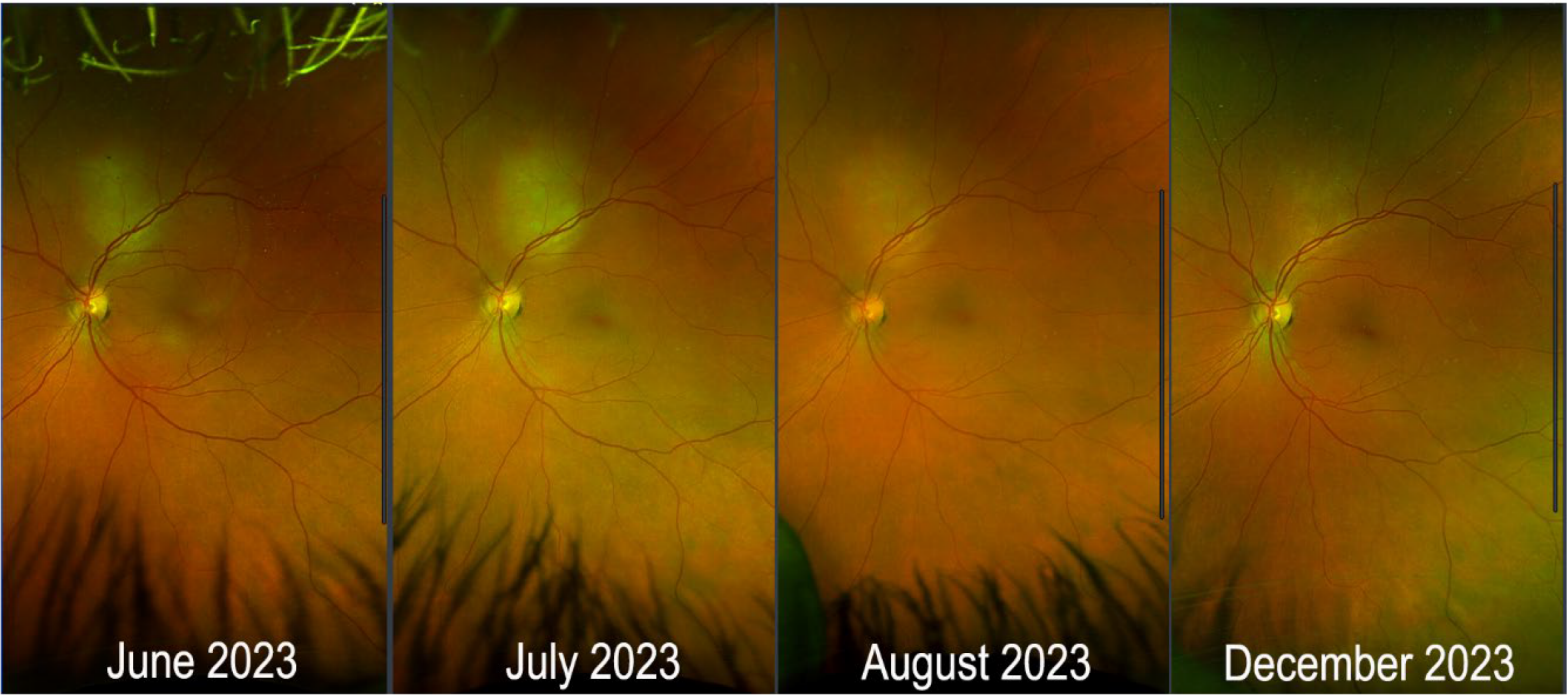
Color fundus photos images. Mass superior to optic nerve with serous detachment involving the macula. L-R: June 2023, July 2023, August 2023, December 2023.

**Figure 3:**
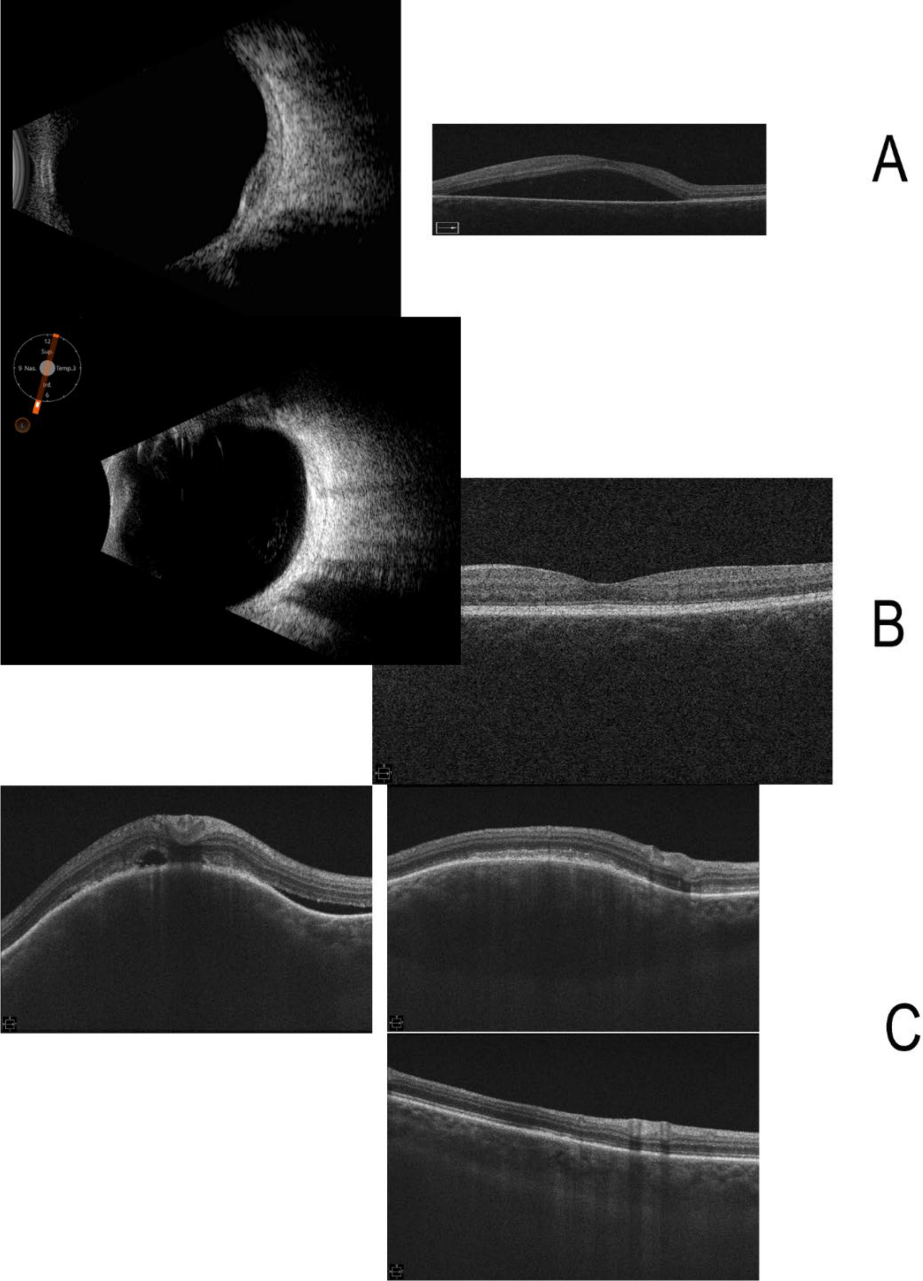
OCT and ophthalmic ultrasound images. **(A)** Initial B-scan ultrasound showing choroidal metastasis. **(B)** Last treatment timepoint -- B-scan ultrasound and macula. **(C)** Optical coherence tomography (OCT) showing cross-section through the choroidal metastasis and retina. The retinal detachment resorbed, and the choroidal metastasis showed good progressive regression.

**Figure 4:**
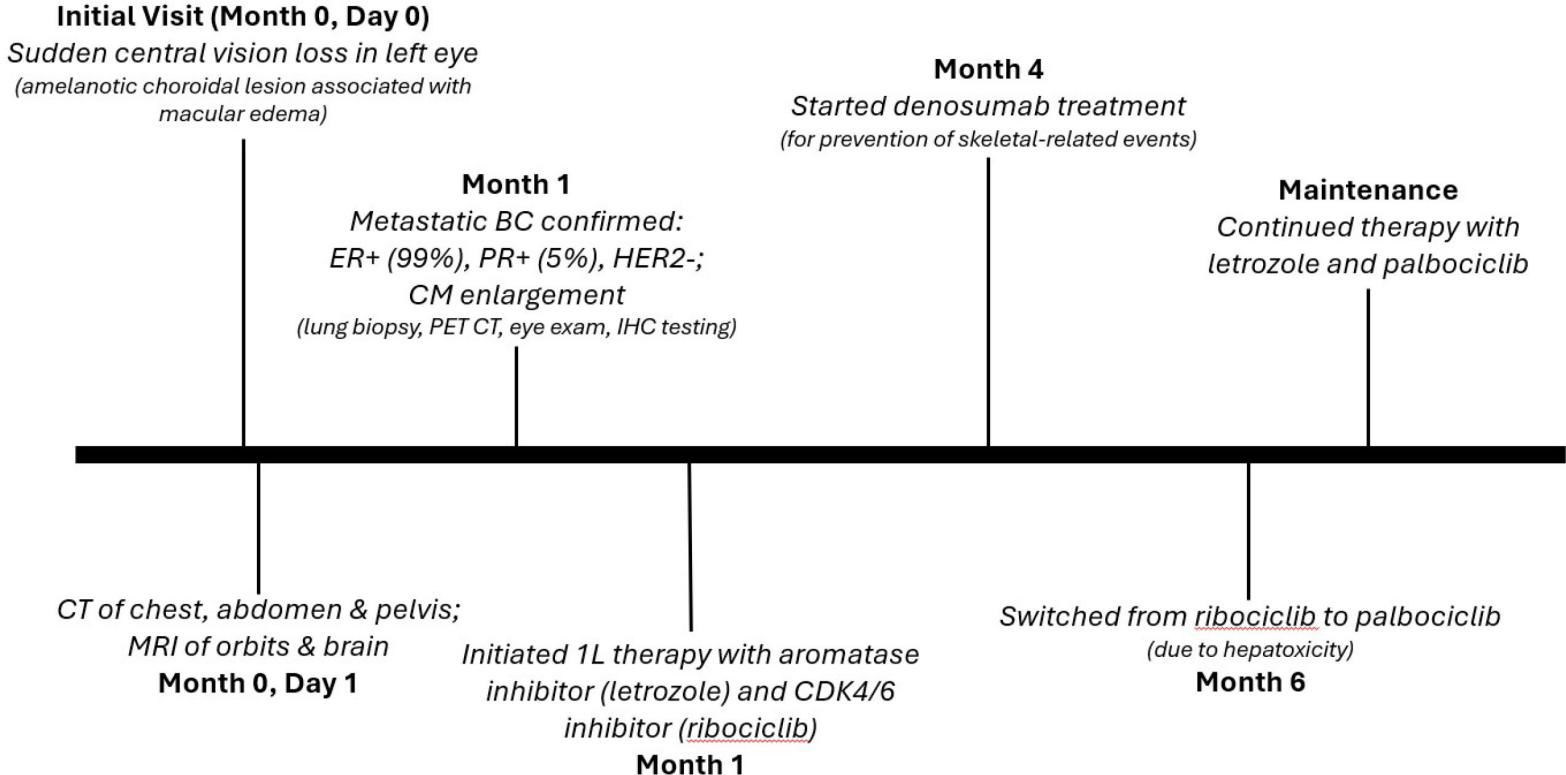
Case report timeline. CM, choroidal metastasis; CT, computed tomography; MRI, magnetic resonance imaging; BC. breast carcinoma; 1L, first line; CDK4/6, cyclin-dependent kinase 4 and 6; PET CT, positron emission tomography CT; IHC, immunohistochemistry.

A CT-guided biopsy of a lung nodule confirmed metastatic breast carcinoma. Immunohistochemical (IHC) studies revealed that the carcinoma was ER+ (99%), PR+ (5%), and HER2 equivocal. Fluorescent *in situ* hybridization (FISH) was negative for amplification of the HER2 gene. A fluorodeoxyglucose positron emission tomography (FDG-PET) combined with CT scan demonstrated a pulmonary nodule with a maximum standardized uptake value (SUV) of 2.5, a subcarinal lymph node with a maximum SUV of 4.4, and a left pubic bone lesion with a maximum SUV of 7. An ophthalmologic examination, with color fundus photos (**Figure 2**), optical coherence tomography (OCT), and ophthalmic ultrasound (**Figure 3**), confirmed CM with serous retinal detachment in the left eye. The right eye was unaffected. A 36-gene panel genetic test was performed and was negative. A next-generation sequencing (NGS) DNA test showed no targetable mutations. A timeline of the patient’s treatment is shown in **Figure 4**.

## 3 Treatment

The patient was initiated on first-line therapy with an aromatase inhibitor (letrozole) and a CDK4/6 inhibitor (ribociclib) (6). Additionally, the patient received bone anti-resorptive therapy with denosumab to prevent skeletal-related events. Ribociclib was held after 1 cycle due to grade 3 hepatotoxicity. The patient was then switched to palbociclib, which was well tolerated with no further treatment delays.

Ophthalmologic follow-up 1 month after initiation of treatment showed an initial enlargement of the lesion with a small improvement in subretinal fluid, followed by dramatic regression of the CM and resolution of the subretinal fluid the following month. MRI of the orbits similarly confirmed resolution of the left globe lesion, and the patient continues to improve clinically.

The patient continues first-line endocrine therapy—palbociclib and letrozole—with good clinical, radiographic, and ophthalmic response. The patient’s most recent nuclear whole-body bone scan and CT scans of her chest, abdomen, and pelvis showed stable disease. Ophthalmologic evaluation confirmed excellent tumor regression on ophthalmic exam and imaging with no new lesions. The patient continues to follow up with oncology and ophthalmology.

## 4 Discussion

The main goal of CM treatment is vision preservation. Treatment options include systemic therapy as well as local interventions. While the gold standard for CM treatment is external beam radiotherapy, systemic medical therapy may be appropriate as a primary treatment, depending on factors such as cancer type, the likelihood of response to systemic treatment, and the extent of ocular disease and symptoms. Other local treatment modalities include photodynamic therapy and adjunctive intravitreal anti-vascular endothelial growth factor (VEGF), which are more commonly used in patients with poor performance status and limited life expectancy (7).

The choroid of the eye exhibits high vascular permeability. This property allows significant penetration of cancer-directed treatment. Since metastatic ER+/HER2- breast cancers respond very well to first-line endocrine therapy with a CDK4/6 inhibitor, it may be appropriate to defer the use of local therapies until CM progression. In our case, the patient had a very rapid response to first-line endocrine therapy with regression of her CM, which emphasizes the important role of a CDK4/6 inhibitor plus endocrine therapy as first-line treatment, even in cases of visceral involvement. CM in breast cancer indicates a poor prognosis and is associated with a high risk of central nervous system (CNS) involvement, significantly impacting the mortality and quality of life of affected patients. Based on the patient’s good response, we decided to save radiation therapy as a future treatment option if needed.

We conducted a literature search in PubMed to identify relevant case reports and case series published since 2006 (**Table 1**). We found a total of 27 cases: 25 female and 2 male patients diagnosed with late-onset CM originating from breast carcinoma. All patients had a history of HR+, HER2- breast cancer and developed ocular metastases several years after initial diagnosis. The latency period between primary breast cancer diagnosis and CM development varied significantly. In most cases, CM diagnosis was identified 2.5 years to over 25 years after the initial cancer diagnosis. In many cases, CM was the first clinical sign of systemic recurrence, underscoring the need for long-term vigilance in breast cancer survivors. Across all cases, systemic endocrine therapy was the main treatment, with consistently favorable outcomes. Among the reviewed cases, local therapy was infrequently employed. In addition to endocrine therapy, 1 patient received external beam radiation therapy, and another was treated with intravitreal bevacizumab. Both patients had favorable clinical responses. Systemic endocrine therapies such as aromatase inhibitors with or without gonadotropin-releasing hormone (GnRH) agonists (used to induce ovarian function suppression in premenopausal women) and CDK4/6 inhibitors or selective ER modulators were the predominant treatment modalities across cases. Local treatments such as radiotherapy were selectively applied at some point of their treatment trajectories, typically in cases with incomplete systemic control or to alleviate local ocular symptoms.

**Table 1:** Cases of choroidal metastases secondary to ER+/HER2- breast cancer.

Author	Patient(s)	Year	Time between primary breast cancer diagnosis & choroidal metastases diagnosis	Systemic treatment of choroidal metastases	Local treatment	Results
Our case	57-year-old female	2025	29 years	Palbociclib and letrozole	N/A	Regression
Fu et al. (8)	39-year-old male	2022	12 years	Palbociclib and goserelin	N/A	Regression
Barke et al. (9)	50s -year-old female	2022	N/A	Abemaciclib and fulvestrant	N/A	Regression
Parakh et al. (10)	83-year-old-female	2022	3 years	Palbociclib and letrozole	N/A	Regression
Levison et al. (11)	Case 1: 73-year-old female; Case 2: 74-year-old female; Case 3: 87-year-old female	2016	All cases range between 20–25 years (median: 22 years)	Case 1: Anastrozole, Case 2: Tamoxifen, then with anastrozole, Case 3: Letrozole	N/A	Regression
Zako et al. (12)	51-year-old female	2012	16 years	Tamoxifen and cyclophosphamide hydrate then letrozole	Bevacizumab	Complete regression
Cancino et al. (13)	50-year-old female	2011	16 years	Letrozole	N/A	Complete regression
Venkatesh et al. (14)	32-year-old female	2007	2.5 years	Letrozole	Radiotherapy	Complete regression
Shome et al. (15)	55-year-old male	2007	5 years	Tamoxifen	N/A	Regression
Manquez et al. (16)	17 cases of females, mean age: 58 years (median: 60; range: 56-80)	2006	Interval:7.6 years (range: 1 month-17 years)	Aromatase inhibitors	N/A	10/17 (59%) patients with ER+ breast cancer responded dramatically to aromatase inhibitors; 7 patients didn’t show regression

The largest case series included in this review (16) reported on 17 female patients with a mean interval of 7.6 years (range: 1 month-17 years) between initial breast cancer diagnosis and CM. In this cohort, 59% of patients with ER+ tumors demonstrated significant regression following treatment with aromatase inhibitors. Collectively, these findings support the efficacy of systemic endocrine therapy as a first-line treatment for CM in patients with HR+, HER2- breast cancer.

We believe it may be appropriate to treat choroidal metastases secondary to ER+, HER2- breast cancer with endocrine therapy in combination with CDK4/6 inhibitors and delay the use of local treatments until progression of CM.Large-scale clinical trials will likely not be feasible due to the low frequency of CM, which may require urgent or emergent therapy.

In the absence of large evidence-based studies, a multidisciplinary approach is always advised for the management of CM. This case contributes to the growing body of literature supporting the role of endocrine therapy and CDK4/6 inhibitors in the management of metastatic breast cancer, including CM.

## Data Availability

The original contributions presented in the study are included in the article/supplementary material. Further inquiries can be directed to the corresponding author.
